# Mental Health of Parents and Preschool-Aged Children During the COVID-19 Pandemic: The Mediating Role of Harsh Parenting and Child Sleep Disturbances

**DOI:** 10.3389/fpsyt.2021.746330

**Published:** 2021-11-29

**Authors:** Peiyao Wang, Xiaoning Sun, Wen Li, Zijing Wang, Shan He, Feng Zhai, Yuan Xin, Linlin Pan, Guanghai Wang, Fan Jiang, Jie Chen

**Affiliations:** ^1^Department of Otolaryngology, Shanghai Children's Medical Center, School of Medicine, Shanghai Jiao Tong University, Shanghai, China; ^2^Department of Developmental and Behavioral Pediatrics, Shanghai Children's Medical Center, School of Medicine, Pediatric Translational Medicine Institute, Shanghai Jiao Tong University, Shanghai, China; ^3^Ministry of Education-Shanghai Key Laboratory of Children's Environmental Health, Shanghai, China

**Keywords:** parental well-being index, child mental health problems, childhood adversity experience, child sleep problems, COVID-19 pandemic, preschoolers

## Abstract

In the context of the coronavirus disease-2019 (COVID-19) pandemic, mental health problems of parents and children have become a public issue. Herein, we explored the association between parental well-being index and child mental health problems during the pandemic and the mediating role of harsh parenting and child sleep disturbances. An online survey was conducted among 16,398 parents of children aged 3–6 years (48.1% girls, M_age_ = 4.69 years, SD_age_ = 0.75 years) from March 15 to 29, 2020. Child mental health (Strengths and Difficulties Questionnaire, SDQ), sleep problems (Children's Sleep Habits Questionnaire, CSHQ), and parental well-being index (World Health Organization-Five Well-Being Index, WHO-5), and harsh parenting were reported by parents. The results revealed that a higher parental well-being index was associated with lower child mental health problems. Harsh parenting and child sleep problems were significant mediators within the association. This study indicates the association between parental well-being index and child mental health during the pandemic and underlying mechanism, and has important implications for reducing parental and child mental health problems.

## Introduction

The coronavirus disease-2019 (COVID-19) has spread across the world rapidly. Currently, the pandemic is still ongoing and even deteriorating in some regions and countries. China has effectively controlled the pandemic by implementing measures such as nationwide home confinement and school closure. Approximately 180 million primary and secondary students and 47 million preschool children who were confined at home during the pandemic experienced a sudden change in lifestyle in China (1). Although guidelines, resources, and interventions have been promptly provided (2), increasing concerns are emerging regarding the mental health of children during the pandemic. Thus, exploring the factors influencing child mental health to ensure the healthy growth of children during the pandemic becomes especially important. Mental health is recognized as the primary factor in having a good quality of life, and happy and confident children are more likely to maintain it into adulthood, thus providing resilience in the face of adversity (3). It is an important factor that affects physical health. For example, studies have demonstrated associations between emotional and behavioral problems and diet quality (4). Consequently, considering the important role of child mental health and the negative impact of the COVID-19 pandemic, it is of critical importance to focus on the factors affecting child mental health during the pandemic.

The strike of the COVID-19 outbreak accompanied by home confinement, social isolation, unhealthy lifestyle, and unfavorable family environment factors have been regarded as an adverse experience that could impair the child mental health (5). Does this mean that lower well-being index of parents and worse parenting style and unhealthy lifestyle, such as child sleep hygiene problems act on child mental health? However, their unique roles during the pandemic have attracted limited attention. Therefore, this study examined the association between parental well-being index and child mental health by using a sample of isolated Chinese preschool-aged children during the COVID-19 pandemic. Furthermore, we examined the internal mechanism of the above relationship.

### Parental Well-Being Index and Child Mental Health Problems

The effect of home confinement on parental and child mental health is of great concern. Epidemiological studies have shown that children exposed to pandemics are particularly vulnerable to behavioral problems, including hyperactivity, conduct disorders, externalizing problems, and general psychological distress (1, 6, 7). Significant disruptions in family lifestyle during the home confinement, combined with heightened stress and anxiety can lead to psychological difficulties in preschoolers.

In the meantime, just like their children, adults face all kinds of stress. In particular, parents have to cope with social distancing and changes in their daily routine, such as working remotely or facing unemployment, combined with additional caring for their children during school hours. In some cases, the changes brought about by the confinement were accompanied by reduced income and new responsibilities, which likely exacerbated the pre-existing difficulties and pressures (8, 9). Yet, despite a growing focus on the impact of COVID-19 on adults' and children's mental health, few studies have explored the association between parental well-being index and child mental health problems in the context of the pandemic. Therefore, based on previous evidence, we propose the following hypothesis in the pandemic:

Hypothesis 1: parental well-being index would be associated with child mental health problems.

### The Mediating Roles of Harsh Parenting and Child Sleep Disturbances

Parenting style refers to a series of practices that create an emotional environment and influence child development and well-being (10). Previous studies have reported that parents' mental health likely affects their parenting behaviors, with harsh parenting style, in turn, leading to child mental health problems (11). In the context of the COVID-19 pandemic, parents experiencing elevated levels of cumulative stress are even more likely to display more rigid and abusive parenting behaviors (12). Ineffective parenting practices are positively associated with child behavioral problems (13). In particular, parental verbal aggression alone as separate and distinct from physical punishment contributes to lower self-esteem in children (14). Concerning parenting strategies, the American Academy of Pediatrics recommends against physical and verbal punishment of children in favor of more effective disciplines for raising healthy children (15). Therefore, increased attention should be paid to investigating the mediating role of parenting style within the parent-child mental health association. Thus, the following hypothesis is developed in relation to the pandemic:

Hypothesis 2: harsh parenting would mediate the relationship between parental well-being index and child mental health problems.

Sleep disturbances frequently occur in preschool children and are associated with a range of adverse health outcomes (16). During the pandemic, the confined preschoolers were reported to demonstrate changes in sleep patterns characterized by later bedtimes and wake times, longer nocturnal, and shorter nap sleep durations (17). Increasing research has indicated the impact of parental psychological functioning on sleep quality in young children. For example, parental depression and stress were linked to child sleep disturbances (18). Moderate/severe maternal depression symptoms were associated with increased odds of children aged 4–5 years sleeping <10 h/day (19). Meanwhile, it has been well-documented that sleep disturbances are associated with poor mental health in children. For example, children with sleep disturbances show more maladaptive emotional generation and regulation processes, more inattention, aggressive and hyperactivity-related problems, more peer problems and school readiness problems (20). Based on these findings, it can be inferred that sleep problems may increase child mental health problems. Thus, we propose the following:

Hypothesis 3: child sleep problems would mediate the relationship between parental well-being index and child mental health problems.

In summary, harsh parenting and child sleep problems are important links that connect parental well-being index and child mental health problems. Environmental and lifestyle factors can affect sleep quality and quantity and lead to sleep disorders (21). Children who live in families with harsh parenting are significantly associated with lower verbal skills and increased behavioral problems, including internalization, externalization, and sleep problems (22). A prospective study demonstrated that hostile parenting predicts child sleep problems (23). Thus, harsh parenting style may be positively contributed to child sleep disturbances. Based on the above, parents with a low well-being index may implement rigid parenting behaviors, which further increase the odds of child sleep disturbances and enhance their mental health problems, a serial link demanding a systematic investigation. Thus, the following hypothesis is proposed:

Hypothesis 4: parental well-being index would be associated with child mental health problems through the serial mediating roles of harsh parenting and child sleep problems.

## Materials and Methods

### Participants and Procedures

The current study used data from a population-based online survey that included parents (including other caregivers) of children aged 3–6 years from 28 provinces across mainland China. Specifically, 25.7% of the questionnaires came from East China (Shanghai, Zhejiang, Jiangsu, and so on), 73.3% from southwest China (Chongqing, Sichuan, Guizhou, and so on), and 1% from other regions (Guangdong, Shanxi, Henan, and so on). The survey was conducted through the WeChat-based survey program, Questionnaire Star (https://www.wjx.cn/), a frequently used online survey platform in China, from March 15 to 29, 2020 when the families were on mandatory home confinement while filling the survey (24). The survey was administered through a combination of non-probabilistic convenience and snowball sampling. The scan code and link for the survey were posted to the public. The parents who agreed to participate were encouraged to forward the survey link to other parents. Participants endorsed their consent before moving on to the survey questions. To promote participation and data quality, a free online workshop entitled “how to help your child get sound sleep during COVID-19?” was delivered to the participants by a psychologist and sleep specialist (GW). We also used a series of logical algorisms in the program to maximize the accuracy and quality of the online survey, such as restricting responses range and de-identity. The participant could submit the questionnaire only after all items were completed to reduce the possibility of accidentally skipping items. According to the logged-in WeChat account, each participant was only allowed to participate once. Since the self-report questionnaires might be affected by participants' response bias, all participants remained anonymous and participated voluntarily.

**Figure 1:**
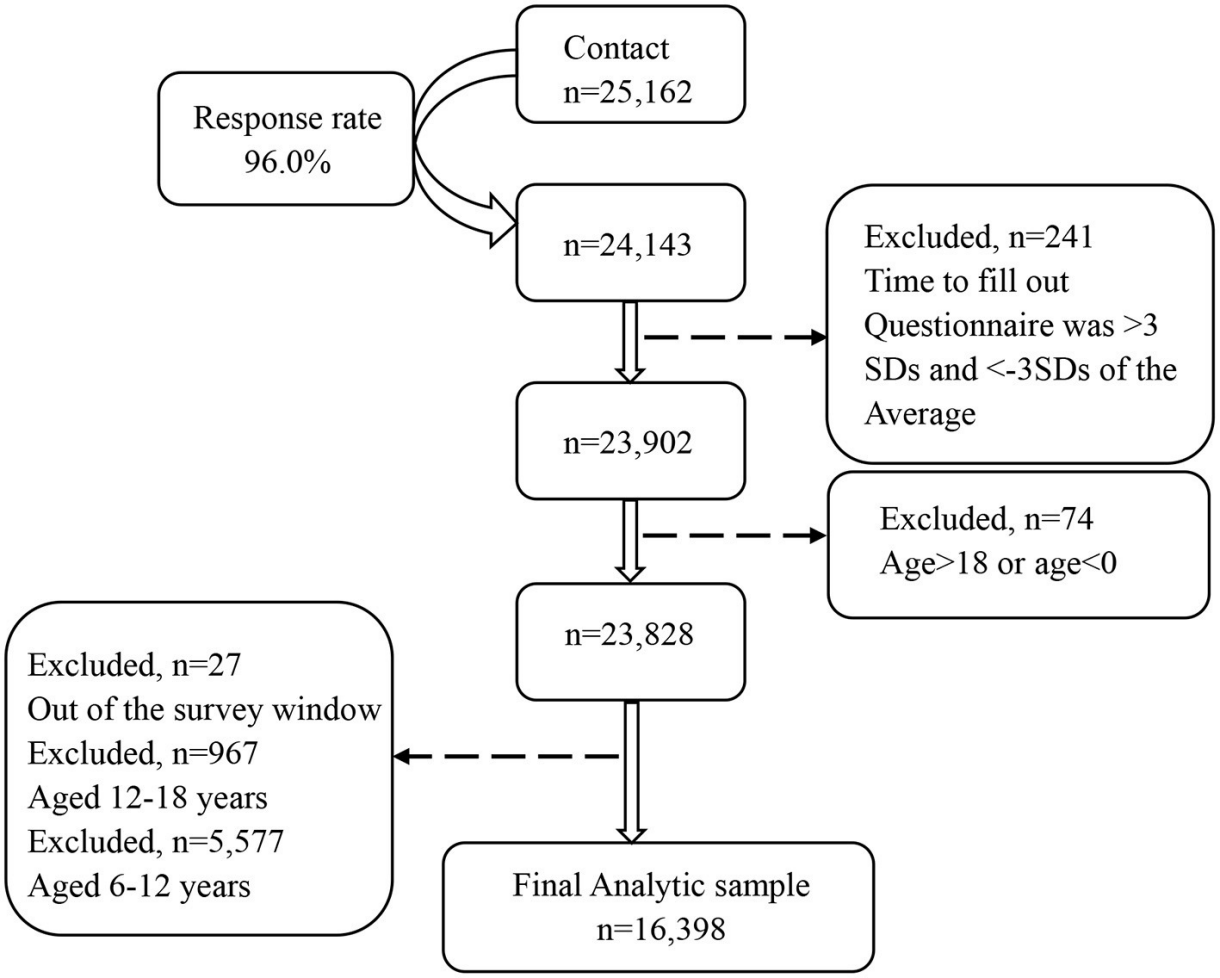
Flow-chart for participants.

Among 25,162 initial contacts, parents of 24,143 children consented to participate, yielding a 96.0% response rate (Figure 1). As we aimed to recruit a general and representative sample of the general population for assessment of child mental health problems during the pandemic, we did not screen and exclude those with suspected COVID-19 infection. We excluded 241 questionnaires with a completion time outside three standard deviations (*M* = 18.87 min, *SD* = 10.56 min) and 7,504 questionnaires outside the age range of 3–6 years old.

### Measures

The survey consisted of a set of sociodemographic questions investigating children's age, gender, presence of siblings, family type (e.g., nuclear family, extended family, etc.), primary caregivers, and parental education level. The primary caregiver was defined as the child's main caretaker and was dichotomized into parental care vs. non-parental care. Parental education level was self-reported on four levels (undergraduate and above, junior college, high school or technical secondary school, and middle school and below). Following this, a series of standardized measures on child mental health, parental well-being index, and sleep problems were presented. Measures on physical activity and screen exposure were also included.

Child mental health problems were evaluated using the Strengths and Difficulties Questionnaire (SDQ). Parents were asked to rate their child's behaviors over the past 2 months on 25 items, compositing five subscales: peer problems, conduct disorders, hyperactivity, emotional problems, and prosocial behavior. We used the SDQ total difficulty score (excluding the prosocial behavior items), with a score of ≥14 indicating mental health problems (25). The Cronbach's α for SDQ was 0.73 and the Kaiser-Meyer-Olkin (KMO) measure of sampling adequacy was 0.89 (*p* < 0.001, Bartlett's test of sphericity) in the current sample.

The World Health Organization-Five Well-Being Index (WHO-5) was used to assess the parental well-being index over the past 2 weeks (26). On five mental health-related statements, parents were asked to rate the frequency on a six-point Likert scale (0 = Never to 5 = All the time). A total score was calculated, with a score of <13 indicating mental health problems (27). The Cronbach's α was 0.92 and the KMO measure of sampling adequacy was 0.89 (*p* < 0.001, Bartlett's test of sphericity) in the current sample.

Harsh parenting was evaluated by two questions: (1) to discipline and regulate a child's behavior, how many times have you physically punished your child (spanking or hitting) without hurting him/her or leaving bruises or marks? (2) To discipline and regulate a child's behavior, how many times have you scolded your child (yelling, shouting, or using words to humiliate him or her)? Parents were required to report the frequency of each statement on a 5-point scale (never, 1 or 2 times per week, 3 or 4 times per week, 5 or 6 times per week, almost every day).

The Children's Sleep Habits Questionnaire (CSHQ), which was used to assess child sleep disturbances over the past week (28), is a standardized and internationally recognized instrument consisting of 33 items covering eight domains: bedtime resistance, sleep anxiety, sleep onset delay, sleep duration, night waking, parasomnia, daytime sleepiness, and sleep-disordered breathing. Parents rated the frequency of each item occurring in their children over the past week on a 3-point scale (usually, sometimes, rarely). A total score >41 indicates global sleep disturbances. The Cronbach's α was 0.73 and the KMO measure of sampling adequacy was 0.84 (*p* < 0.001, Bartlett's test of sphericity) in the current sample.

Daily time spent on physical activity over the past week was reported as <30 min, 30–60 min, >60 min. Daily exposure to media over the past month was reported on weekdays and weekends. The average daily screen exposure time = (screen exposure time on weekdays^*^5 + screen exposure time on weekends^*^2)/7. As previous studies have linked physical activity and screen exposure time to child mental health problems (29–31), they were considered as covariates in the current study.

### Data Analysis

Missing data for all key variables were <1% (*n* = 154) and were handled by listwise deletion. Descriptive analyses were used for sociodemographic data. The multicollinearity test was conducted to exclude multicollinearity problems among the variables. We used Harman's single-factor test to verify the presence of common method bias. We also used Spearman correlations to investigate associations between parental well-being index, harsh parenting, child sleep problems, and child mental health problems. We calculated the Cronbach's alpha to show the reliability of the measures and used the Kaiser-Meyer-Olkin (KMO) test to show its validity. Data analysis was conducted using SPSS 26.0. Finally, we employed PROCESS Model 6 in SPSS 26.0 to examine Hypotheses 1 to 4. In addition, a 95% bias-corrected confidence interval with 5,000 bootstrap samples was applied to determine the significance of the mediational effect.

## Results

### Sample Characteristics

Descriptive data are summarized in Table 1. A total of 16,398 children aged 3–6 years (*M* = 4.69 years, *SD* = 0.75 years) were finally included in the study. The demographic survey results showed that participants comprised 8,512 boys (51.9%) and 7,886 girls (48.1%). Of the children, 58.0% had at least one sibling. Family type distribution was 42.4% with a nuclear family, 54.5% with extended family, 2% with single-parent family, and 1.1% with other family types. The primary caregiver of the children was mostly the parent (71.1%), while for the remaining 28.9% of children, it was a grandparent or others. There were 36.7% parents with the highest level of undergraduate education and above, 22.3% with junior college, 26.7% with high school or technical secondary school, and 24.3% with the lowest level of middle school or below. In 56.5% of the preschoolers, physical activity was <30 min/day, in 35.8%, it was 30–60 min/day, and in 7.7%, it was more than 60 min/day. The average screen time among preschoolers was 2.05 ± 1.87 h/day.

**Table 1:** Descriptive characteristics of the sample (*n* = 16,398).

**Variables**	**Total sample**
**Child age (years)**	
Mean (SD)	4.69 ± 0.75
**Child gender**, ***n*** **(%)**	
Male	8,512 (51.9)
Female	7,886 (48.1)
**Child siblings**, ***n*** **(%)**	
One or more	9,503 (58.0)
None	6,895 (42.0)
**Child family type**, ***n*** **(%)**	
Nuclear family	6,850 (42.4)
Extended family	8,944 (54.5)
Single-parent family	324 (2.0)
Others	180 (1.1)
**Child primary caregiver**, ***n*** **(%)**	
Parental care	11,659 (71.1)
Grandparents or others	4,739 (28.9)
**Child physical activity**, ***n*** **(%)**	
<30 min	9,266 (56.5)
30–60 min	5,868 (35.8)
>60 min	1,264 (7.7)
**Child screen exposure time (hours)**	
Mean (SD)	2.05 ± 1.87
**Parental education**, ***n*** **(%)**	
Middle school and below	3,982 (24.3)
High school or technical secondary school	4,375 (26.7)
Junior college	3,657 (22.3)
Undergraduate and above	4,056 (36.7)
Others	328 (2.0)

### Multicollinearity Test and Common-Method Bias Test

The multicollinearity test revealed that the tolerance of each variable was between 0.90 and 0.92, and the variance expansion factor ranged between 1.09 and 1.11. These findings revealed no multicollinearity problem among the variables.

The results of Harman's single-factor examination showed that eight factors had eigenvalues >1, and the first factor explained 11.89% of the total variance. This result did not exceed the critical value of 40% (32). The above methods indicate an absence of serious common method bias in the current study.

### Correlations Among Primary Variables

The Spearman correlations (Table 2) showed that significant but moderate positive relationships between child mental health problems with harsh parenting (*r* = 0.27, *p* < 0.01) and child sleep problems (*r* = 0.34, *p* < 0.01), as well as between harsh parenting and child sleep problems (*r* = 0.20, *p* < 0.01). There were significant but moderate negative relationships between parental well-being index and harsh parenting (*r* = −0.25, *p* < 0.01), child sleep problems (*r* = −0.26, *p* < 0.01), and child mental health problems (*r* = −0.23, *p* < 0.01).

**Table 2:** Means, standard deviations, correlations, and reliabilities (in brackets).

	**M**	**SD**	**1**	**2**	**3**	**4**	**5**	**6**
1 Physical activity	1.51	0.64	-					
2 Screen exposure time	2.05	1.87	0.06^**^	-				
3 Parental well-being index	15.99	5.56	0.08^**^	−0.10^**^	-			
4 Harsh parenting	3.73	1.75	0.03^**^	0.10^**^	−0.25^**^	-		
5 Child sleep problems	43.34	6.52	−0.04^**^	0.12^**^	−0.26^**^	0.20^**^	-	
6 Child mental health problems	18.66	4.79	0.03^**^	0.11^**^	−0.23^**^	0.27^**^	0.34^**^	-

*p < 0.05,

** p < 0.01, N = 16,398 for preschool children, physical activity (“1” < 30 min; “2” 30–60 min; “3” > 60 min), The average daily screen exposure time = (screen exposure time on weekdays*5+ screen exposure time on weekends*2)/7.

### Serial Mediation Model

**Figure 2:**
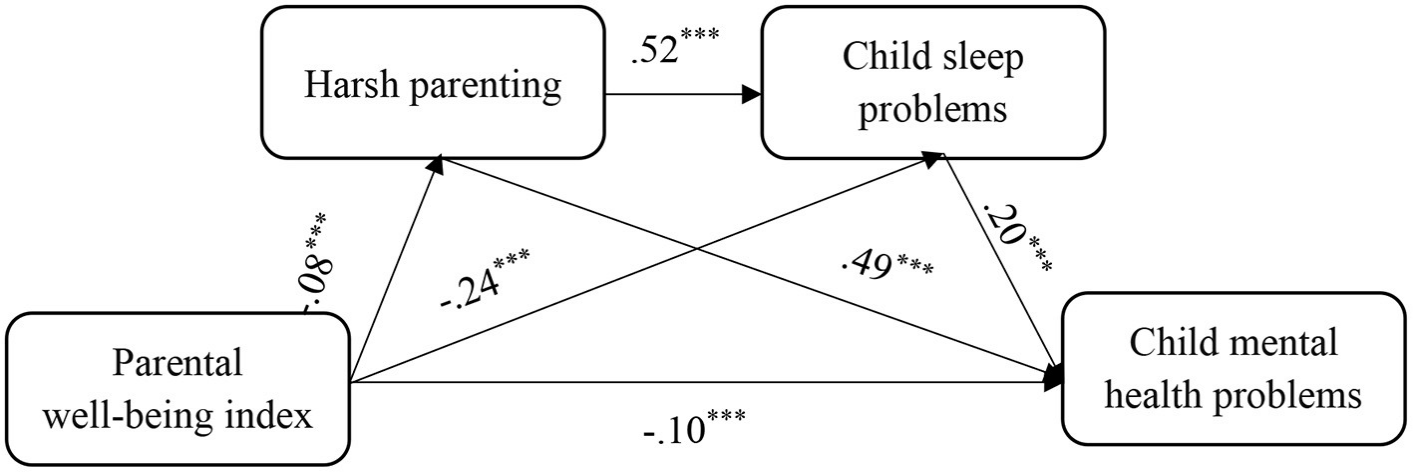
Roadmap of the influence of parental mental health on child mental health. ***p < 0.001.

Table 3 and Figure 2 show that the direct effect of parental well-being index on child mental health problems was significant and negative (β = −0.10, *p* < 0.001). In the path of “parental well-being index → harsh parenting → child mental health problems,” parental well-being index had a significant negative impact on harsh parenting (β = −0.08, *p* < 0.001), which in turn had a significant positive impact on child mental health problems (β = 0.49, *p* < 0.001). In other words, the parental well-being index enhanced the children's psychological health by reducing harsh parenting. In the path of “parental well-being index → child sleep problems → child mental health problems,” parental well-being index had a significant negative impact on child sleep problems (β = −0.24, *p* < 0.001), which in turn had a significant positive impact on child mental health problems (β = 0.20, *p* < 0.001). Thus, the parental well-being index improved child psychological health problems by weakening child sleep problems. Finally, serial mediation analysis revealed that in the path of “parental well-being index → harsh parenting → child sleep problems → child mental health problems,” harsh parenting had a significant positive impact on child sleep problems (β = 0.52, *p* < 0.001). This indicated that the parental well-being index reduced child sleep problems by alleviating the harsh parenting, which in turn improved child mental health problems.

**Table 3:** Model coefficients for the serial mediation analysis.

**Variables**	**Model 1**			**Model 2**			**Model 3**		
	**Harsh parenting**			**Child sleep problems**			**Child mental health problems**		
	**B**	**SE**	**T**	**B**	**SE**	**t**	**B**	**SE**	**T**
Screen exposure time	0.06	0.01	7.98^***^	0.28	0.03	10.49^***^	0.09	0.02	5.02^***^
Physical activity	0.15	0.02	7.00^***^	−0.23	0.08	−3.03^**^	0.28	0.05	5.11^***^
Parental well-being index	−0.08	0.00	−31.48^***^	−0.24	0.01	−26.02^***^	−0.10	0.01	−14.84^***^
Harsh parenting				0.52	0.03	18.01^***^	0.49	0.02	24.06^***^
Child sleep problem							0.20	0.01	35.93^***^
*R* ^2^	0.06			0.09			0.17		
F	374.55^***^			377.59^***^			648.61^***^		

*p < 0.05,

** *p < 0.01*,

*** p < 0.001, N = 16,398 for preschool children, physical activity (“1” < 30 min; “2” 30–60 min; “3” > 60 min), The average daily screen exposure time = (screen exposure time on weekdays*5+ screen exposure time on weekends*2)/7.

Table 4 shows that the direct and total indirect effect of parental well-being on child mental health was −0.10 (95% CI −0.11, −0.08; *p* < 0.001) and −0.09 (95% CI −0.10, −0.09; *p* < 0.001), respectively, with a total effect of −0.19. The mediating effect of parental well-being index on harsh parenting was −0.04, *p* < 0.001, with an effect size of 19.76 percent. The mediating effect of parental well-being index on child mental health problems through sleep problems was −0.05, *p* < 0.001, with an effect size of 24.84 percent. The mediating effect of parental well-being index on child mental health problems through harsh parenting and sleep problems was −0.01, *p* < 0.001, with an effect size of 4.13 percent.

**Table 4:** Breakdown table of the total, direct, and mediating effects.

**Parental mental health**	**Effect**	**Boot SE**	**Boot LLCI**	**Boot ULCI**	**Effect ratio %**
Direct effects	−0.10	0.01	−0.11	−0.08	51.32
Total indirect effects	−0.09	0.00	−0.10	−0.09	48.68
Indirect effect 1	−0.04	0.00	−0.04	−0.03	19.76
Indirect effect 2	−0.05	0.00	−0.05	−0.04	24.84
Indirect effect 3	−0.01	0.00	−0.01	−0.01	4.13

*The path of indirect effect 1 is “parental well-being index → harsh parenting → child mental health problems,” the path of indirect effect 2 is “parental well-being index → child sleep problems → child mental health problems,” and the path of indirect effect 3 is “parental well-being index → harsh parenting → child sleep problems → child mental health problems.”*

## Discussion

To our best knowledge, the current study is among the first to explore the relationship between parental well-being index and child mental health problems, and the mediating role of harsh parenting and child sleep problems in the context of COVID-19 pandemic. Our findings showed that the parental well-being index had an indirect and negative effect on child mental health problems. Additionally, separately, harsh parenting and child sleep problems mediated the relationship between parental well-being index and child mental health problems. More importantly, a significant serial mediation was identified: parents with low well-being index tended to exert more toward harsh parenting, which further increased child sleep problems and, subsequently, resulted in mental health problems in children.

The current study corroborated the negative association between parental well-being index and child mental health problems in the context of the COVID-19 pandemic. This finding is consistent with a previous study reporting that mental distress among caregivers was associated with an increased risk of child health issues during the pandemic (33). It is well-known that, due to their cognitive and psychological immaturity, children are particularly vulnerable to stress, in the incidence of natural disasters (34). An emotionally and physically stable parent is more capable of helping children buffering their stress and managing their negative feeling (35). However, in parents who increasingly experience a reduction in income, changes in daily routine, and new responsibilities during the pandemic, their ability to comfort children inevitably diminishes over time, thus increasing the risk of trauma to the pandemic in children and youth, resulting in enduring emotional consequences. Research shows that children as young as 2 years are aware of the changes around them (36). Therefore, observing poor well-being in parents, such as high anxiety and depressive symptoms may lead to a higher likelihood of children suffering from mental health problems.

The current results revealed the mediating roles of harsh parenting and child sleep problems between parental well-being index and child mental health problems during the pandemic. Indeed, as parental stress levels rise over the pandemic period, parents may be more likely to exert harsh parenting (11). Specifically, previous studies have shown that the rates of child abuse, neglect, and exploitation are likely to exacerbate during such stressful periods (37). Our findings are consistent with recent research showing that parental distress and social isolation are important risk factors contributing to child abuse and neglect, domestic violence, and a deterioration of the parent-child relationship (38–40). Additionally, children whose parents were, in general, more pessimistic displayed worse emotion regulation and were more likely to show externalizing behaviors than children whose parents were warmer and more frequently expressed positive emotions (41). Verbal abuse during childhood has been associated with the externalization and internalization of various disorders in adulthood (42, 43). Therefore, our findings have significant implications for promoting a positive family environment during the pandemic.

Our results further revealed that sleep problems might constitute a pathway for the relationship between parental well-being index and child mental health. Child sleep problems were responsible for more than 20 percent of the parental well-being index related to child mental health problems, which was stronger than the other two indirect effects, indicating that parental well-being index affected child mental health problems severity mainly through sleep. Excessive parental involvement at bedtime were associated with increased child nocturnal awakenings (44). During the lockdown, mothers may be excessively vigilant to their children's health and sleep, and thus overestimating the children's sleep problems. Whereas, Gregory and Sadeh (45) have proposed that the association between sleep and mental health may be bidirectional (46), it is also possible that changes in sleep during the COVID-19 pandemic contribute to or even exacerbate psychological health (5). Since sleep problems in children potentially have a significant impact on certain aspects of the child's psychological health, including cognitive development and emotional/behavioral development, prioritizing interventions to maintain the child's quality of sleep are strongly encouraged.

In the present study, we also examined the association between harsh parenting and child sleep problems during home confinement, thus accounting for the lack of evidence on this topic. The impact of COVID-19 and lockdown measures can increase parenting stress, which in turn could increase the use of harsh parenting and harm the relationship between parents and their children (47). Previous studies reported that adverse childhood experiences like child abuse, neglect, and poor family environment experienced before age 18 were positively associated with a variety of sleep problems in adults, including insufficient sleep, insomnia, and nightmares (48). The proposed underlying mechanisms include childhood adversities that may increase corticotropin-releasing hormone (CRH) reactivity and subsequently hypothalamic-pituitary-adrenal (HPA) axis, which in turn affect sleep quality (48). Previous studies have shown that poor sleep quality can induce consequences ranging from inattentiveness, reduction in executive functioning to mood disturbances (49). Insufficient sleep duration in children was associated with mental health disorders (50). Some studies also suggested that the rapid-eye-movement sleep involved in emotional memory processing may be impaired among subjects who were exposed to trauma (51). Therefore, measures could be undertaken to elevate the parental well-being index, which in turn could weaken the mediating role of harsh parenting and child sleep problems, thus reducing child mental health problems.

Our findings have important implications for prioritizing prevention and intervention efforts to reduce and eliminate the influence of poor parental well-being on child mental health problems during and following the pandemic, particularly by promoting positive parenting style and reducing child sleep disturbances. For example, positive parenting, such as effective parent-child communication about pandemics is critical for child mental health and can have short- and long-term protective effects (52, 53). Schools can also actively promote a health-conscious schedule, good personal hygiene, encourage physical activities, appropriate diet, and good sleep habits, and incorporate such health promotion materials into the school curriculum (54). This can not only develop children's self-discipline skills but also reduce the burden of parents. It is the responsibility of all stakeholders to minimize the negative impact during this sudden public health event.

## Limitations, Strengths, and Future Directions

The current study has several strengths. First, as few studies on the effects of parental well-being index on child mental health problems during the COVID-19 epidemic were available at the time of the investigation, the current study furthered our understanding of their association and mediating factors. Second, the online survey in a large-scale and sociodemographic-diverse sample of young Chinese children, and the use of validated measures maximized the generalizability and accuracy of our findings. The present study also has several limitations that should be considered. First, our data did not allow us to differentiate the findings between urban and suburban areas. Second, several exposure variables were measured with one or two self-made items, and future studies would better use standardized measures. Third, no information about previous diagnosis or difficulties of both parents and children was retrospectively collected, and parental reports on the time of data collection might be biased. The potential self-selection bias in the study should also be noted. Parents who were more concerned about their children's mental health were more likely to participate in the study. Finally, although the use of a cross-sectional online survey was considered the optimal way to obtain timely information on the national level, it prevented us from detecting the direction of causality. Future studies should further investigate the mental health of parents and preschoolers after home confinement.

## Conclusion

The current study showed that the parental well-being index was associated with child mental health problems, and the association was mediated by harsh parenting and child sleep problems. Our findings highlight the importance of comprehensive strategies regarding both the parental well-being index and child mental health problems in the context of the COVID-19 pandemic. To promote optimal mental health in young children during the pandemic and thereafter, parental well-being index, positive parenting skills, and healthy sleep habits should be prioritized. The current study inspires further research and discussion concerning parental or child psychological health during adversity such as the COVID-19 pandemic.

## Data Availability Statement

The raw data supporting the conclusions of this article will be made available by the authors, without undue reservation.

## Ethics Statement

The studies involving human participants were reviewed and approved by the Institutional Review Board of Shanghai Children's Medical Center, Shanghai Jiao Tong University School of Medicine (SCMCIRB-W2020042). Written informed consent to participate in this study was provided by the participants' legal guardian/next of kin.

## Author Contributions

PW, GW, FJ, and JC contributed to the concept and the design of the research project. WL, ZW, SH, FZ, YX, and LP contributed to the administration of online surveys and the acquisition of data. PW, XS, WL, GW, FJ, and JC contributed to the analysis, interpretation, and description of data. PW, XS, WL, ZW, GW, FJ, and JC participated in drafting the article and revising it critically for important intellectual content. All authors contributed to the article and approved the submitted version.

## Funding

This study was supported by the National Natural Science Foundation of China (82071493, 81700907), Shanghai Municipal Health Commission Funds (ZHYY-ZXYJHZX-202009), Shanghai Children's Medical Center, Shanghai Jiao Tong University School of Medicine Project 3311 (ZCQ-SCMC2018-9), Shanghai Science and Technology Commission (18JC1420305; 19QA1405800; 19411968800) and Shanghai Pudong District Technology Development Funds (PKJ2018-Y45).

## Conflict of Interest

The authors declare that the research was conducted in the absence of any commercial or financial relationships that could be construed as a potential conflict of interest.

## Publisher's Note

All claims expressed in this article are solely those of the authors and do not necessarily represent those of their affiliated organizations, or those of the publisher, the editors and the reviewers. Any product that may be evaluated in this article, or claim that may be made by its manufacturer, is not guaranteed or endorsed by the publisher.

## References

[1] Leung CC Lam TH Cheng KK. Mass masking in the COVID-19 epidemic: people need guidance. Lancet. (2020) 395:945. 10.1016/S0140-6736(20)30520-1 32142626 PMC7133583

[2] World Health Organization. (2020) WHO Coronavirus Disease (COVID-19) Dashboard. Available online at: https://covid19.who.int/?gclid=EAIaIQobChMIntrH6YLK6QIVQdeWCh0VyApcEAAYASABEgLrXvD_BwE

[3] World Health Organization. (2012) Adolescent Mental Health - Mapping actions of nongovernmental organizations and other international development organizations. Available online at: http://tinyurl.com/j2yzanq

[4] Oddy WH Robinson M Ambrosini GL O'Sullivan TA de Klerk NH Beilin LJ. The association between dietary patterns and mental health in early adolescence. Prev Med. (2009) 49:39–44. 10.1016/j.ypmed.2009.05.009 19467256

[5] Becker SP Gregory AM. Editorial perspective: perils and promise for child and adolescent sleep and associated psychopathology during the COVID-19 pandemic. J Child Psychol Psychiatry. (2020) 61:757–9. 10.1111/jcpp.13278 32474941 PMC7300787

[6] Dray J Bowman J Campbell E Freund M Wolfenden L Hodder RK. Systematic review of universal resilience-focused interventions targeting child and adolescent mental health in the school setting. J Am Acad Child Adolesc Psychiatry. (2017) 56:813–24. 10.1016/j.jaac.2017.07.780 28942803

[7] Clark H Coll-Seck AM Banerjee A Peterson S Dalglish SL Ameratunga S. A future for the world's children? A WHO–UNICEF–Lancet Commission. Lancet. (2020) 395:605–58. 10.1016/S0140-6736(19)32540-1 32085821

[8] Cluver L Lachman JM Sherr L Wessels I Krug E Rakotomalala S. Parenting in a time of COVID-19. Lancet. (2020) 395:e64. 10.1016/S0140-6736(20)30736-4 32220657 PMC7146667

[9] Fegert JM Vitiello B Plener PL Clemens V. Challenges and burden of the Coronavirus 2019 (COVID-19) pandemic for child and adolescent mental health: a narrative review to highlight clinical and research needs in the acute phase and the long return to normality. Child Adolesc Psychiatry Ment Health. (2020) 14:20. 10.1186/s13034-020-00329-3 32419840 PMC7216870

[10] Marchetti D Fontanesi L Di Giandomenico S Mazza C Roma P Verrocchio MC. The effect of parent psychological distress on child hyperactivity/inattention during the COVID-19 lockdown: testing the mediation of parent verbal hostility and child emotional symptoms. Front Psychol. (2020) 11:567052. 10.3389/fpsyg.2020.567052 33362632 PMC7758226

[11] Beckerman M van Berkel SR Mesman J Alink LR. The role of negative parental attributions in the associations between daily stressors, maltreatment history, and harsh and abusive discipline. Child Abuse Negl. (2017) 64:109–16. 10.1016/j.chiabu.2016.12.015 28081496

[12] Liu Y Merritt DH. Familial financial stress and child internalizing behaviors: the roles of caregivers' maltreating behaviors and social services. Child Abuse Negl. (2018) 86:324–35. 10.1016/j.chiabu.2018.09.002 30220424

[13] Salari R Wells MB Sarkadi A. Child behaviour problems, parenting behaviours and parental adjustment in mothers and fathers in Sweden. Scand J Public Health. (2014) 42:547–53. 10.1177/1403494814541595 25005931

[14] Solomon CR Serres F. Effects of parental verbal aggression on children's self-esteem and school marks. Child Abuse Negl. (1999) 23:339–51. 10.1016/S0145-2134(99)00006-X 10321771

[15] Sege RD Siegel BS. Effective discipline to raise healthy children. Pediatrics. (2018) 142:e20183112. 10.1542/peds.2018-3112 30397164

[16] Liu J Zhou G Wang Y Ai Y Pinto-Martin J Liu X. Sleep problems, fatigue, and cognitive performance in Chinese kindergarten children. J Pediatr. (2012) 161:520–5 e522. 10.1016/j.jpeds.2012.03.018 22521112 PMC3404213

[17] Liu Z Tang H Jin Q Wang G Yang Z Chen H. Sleep of preschoolers during the coronavirus disease 2019 (COVID-19) outbreak. J Sleep Res. (2021) 30:e13142. 10.1111/jsr.13142 32716566

[18] El-Sheikh M Kelly RJ. Family functioning and children's sleep. Child Dev Perspect. (2017) 11:264–9. 10.1111/cdep.12243 29731807 PMC5931738

[19] Schultz LF Kroll C Constantino B Trombelli M El Rafihi-Ferreira R Mastroeni MF. Association of maternal depression and anxiety symptoms with sleep duration in children at preschool age. Matern Child Health J. (2020) 24:62–72. 10.1007/s10995-019-02843-z 31823116

[20] Tso W Rao N Jiang F Li AM Lee SL Ho FK. Sleep duration and school readiness of chinese preschool children. J Pediatr. (2016) 169:266–71. 10.1016/j.jpeds.2015.10.064 26608085

[21] Rezaei M Khormali M Akbarpour S Sadeghniiat-Hagighi K Shamsipour M. Sleep quality and its association with psychological distress and sleep hygiene: a cross-sectional study among pre-clinical medical students. Sleep Sci. (2018) 11:274–80. 10.5935/1984-0063.20180043 30746046 PMC6361305

[22] Berthelon M Contreras D Kruger D Palma MI. Harsh parenting during early childhood and child development. Econ Hum Biol. (2020) 36:100831. 10.1016/j.ehb.2019.100831 31816562

[23] Rhoades KA Leve LD Harold GT Mannering AM Neiderhiser JM Shaw DS. Marital hostility and child sleep problems: direct and indirect associations via hostile parenting. J Fam Psychol. (2012) 26:488–98. 10.1037/a0029164 22888782 PMC3824960

[24] Li W Wang Z Wang G Ip P Sun X Jiang Y. Socioeconomic inequality in child mental health during the COVID-19 pandemic: first evidence from China. J Affect Disord. (2021) 287:8–14. 10.1016/j.jad.2021.03.009 33761325 PMC9754677

[25] Goodman R. Psychometric properties of the strengths and difficulties questionnaire. J Am Acad Child Adolesc Psychiatry. (2001) 40:1337–45. 10.1097/00004583-200111000-00015 11699809

[26] WHO Collaborating Centre in Mental Health. (2020). Chinese version of the WHO-Five Well-Being Index. Available online at: http://www.who-5.org

[27] Topp CW Ostergaard SD Sondergaard S Bech P. The WHO-5 Well-Being Index: a systematic review of the literature. Psychother Psychosom. (2015) 84:167–76. 10.1159/000376585 25831962

[28] Owens JA Spirito A McGuinn M. The Children's Sleep Habits Questionnaire (CSHQ): psychometric properties of a survey instrument for school-aged children. Sleep. (2000) 23:1043–51. 10.1093/sleep/23.8.1d 11145319

[29] Domingues-Montanari S. Clinical and psychological effects of excessive screen time on children. J Paediatr Child Health. (2017) 53:333–8. 10.1111/jpc.13462 28168778

[30] Chekroud SR Gueorguieva R Zheutlin AB Paulus M Krumholz HM Krystal JH. Association between physical exercise and mental health in 1·2 million individuals in the USA between 2011 and 2015: a cross-sectional study. Lancet Psychiatry. (2018) 5:739–46. 10.1016/S2215-0366(18)30227-X 30099000

[31] Alves JM Yunker AG DeFendis A Xiang AH Page KA. Associations between affect, physical activity, and anxiety among US children during COVID-19. Preprint. medRxiv [Preprint]. (2020) 10.1101/2020.10.20.20216424 33720550 PMC8250275

[32] Dobbins EGH. Collectivistic orientation in teams: an individual and group-level analysis. J Organ Behav. (1997) 18:275–95.

[33] Horiuchi S Shinohara R Otawa S Akiyama Y Ooka T Kojima R. Caregivers' mental distress and child health during the COVID-19 outbreak in Japan. PLoS One. (2020) 15:e0243702. 10.1371/journal.pone.0243702 33301517 PMC7728265

[34] Galea S Nandi A Vlahov D. The epidemiology of post-traumatic stress disorder after disasters. Epidemiol Rev. (2005) 27:78–91. 10.1093/epirev/mxi003 15958429

[35] Courtney D Watson P Battaglia M Mulsant BH Szatmari P. COVID-19 Impacts on child and youth anxiety and depression: challenges and opportunities. Can J Psychiatry. (2020) 65:688–91. 10.1177/0706743720935646 32567353 PMC7502880

[36] Stein A Dalton L Rapa E Bluebond-Langner M Hanington L Stein KF. Communication with children and adolescents about the diagnosis of their own life-threatening condition. Lancet. (2019) 393:1150–63. 10.1016/S0140-6736(18)33201-X 30894271

[37] Lee J. Mental health effects of school closures during COVID-19. Lancet Child Adolesc Health. (2020) 4:421. 10.1016/S2352-4642(20)30109-7 32302537 PMC7156240

[38] Brown SM Doom JR Lechuga-Pena S Watamura SE Koppels T. Stress and parenting during the global COVID-19 pandemic. Child Abuse Negl. (2020) 110:104699. 10.1016/j.chiabu.2020.104699 32859394 PMC7440155

[39] Patrick SW Henkhaus LE Zickafoose JS Lovell K Halvorson A Loch S. Well-being of parents and children during the COVID-19 pandemic: a national survey. Pediatrics. (2020) 146:e2020016824. 10.1542/peds.2020-016824 32709738

[40] Gassman-Pines A Ananat EO Fitz-Henley J II. COVID-19 and parent-child psychological well-being. Pediatrics. (2020) 146:e2020007294. 10.1542/peds.2020-007294 32764151 PMC7546085

[41] Eisenberg N Losoya S Fabes RA Guthrie IK Reiser M Murphy B. Parental socialization of children's dysregulated expression of emotion and externalizing problems. J Fam Psychol. (2001) 15:183–205. 10.1037//0893-3200.15.2.183 11458628

[42] Carr CP Martins CM Stingel AM Lemgruber VB Juruena MF. The role of early life stress in adult psychiatric disorders: a systematic review according to childhood trauma subtypes. J Nerv Ment Dis. (2013) 201:1007–20. 10.1097/NMD.0000000000000049 24284634

[43] Falgares G Marchetti D Manna G Musso P Oasi O Kopala-Sibley DC. Childhood maltreatment, pathological personality dimensions, and suicide risk in young adults. Front Psychol. (2018) 9:806. 10.3389/fpsyg.2018.00806 29875729 PMC5974613

[44] Sadeh A Tikotzky L Scher A. Parenting and infant sleep. Sleep Med Rev. (2010) 14:89–96. 10.1016/j.smrv.2009.05.003 19631566

[45] Gregory AM Sadeh A. Sleep, emotional and behavioral difficulties in children and adolescents. Sleep Med Rev. (2012) 16:129–36. 10.1016/j.smrv.2011.03.007 21676633

[46] Cellini N Di Giorgio E Mioni G Di Riso D. Sleep and psychological difficulties in italian school-age children during COVID-19 lockdown. J Pediatr Psychol. (2021) 46:153–67. 10.1093/jpepsy/jsab003 33517438 PMC7928801

[47] Chung G Lanier P Wong PYJ. Mediating effects of parental stress on harsh parenting and parent-child relationship during Coronavirus (COVID-19) pandemic in Singapore. J Fam Violence. (2020) 1–12. Advance online publication. 10.1007/s10896-020-00200-1 32895601 PMC7467635

[48] Kajeepeta S Gelaye B Jackson CL Williams MA. Adverse childhood experiences are associated with adult sleep disorders: a systematic review. Sleep Med. (2015) 16:320–30. 10.1016/j.sleep.2014.12.013 25777485 PMC4635027

[49] Owens JA Weiss MR. Insufficient sleep in adolescents: causes and consequences. Minerva Pediatr. (2017) 69:326–36. 10.23736/S0026-4946.17.04914-3 28211649

[50] Xiang S Dong J Li X Li L Boffano P. Association between sleep duration, physical activity, and mental health disorders: a secondary analysis of the national survey of children's health 2017-2018. BioMed Res Int. (2021) 2021:5585678. 10.1155/2021/5585678 33816615 PMC7987431

[51] Cowdin N Kobayashi I Mellman TA. Theta frequency activity during rapid eye movement (REM) sleep is greater in people with resilience versus PTSD. Exp Brain Res. (2014) 232:1479–85. 10.1007/s00221-014-3857-5 24531640 PMC4449337

[52] Clark H Coll-Seck AM Banerjee A Peterson S Dalglish SL Ameratunga S. After COVID-19, a future for the world's children? Lancet. (2020) 396:298–300. 10.1016/S0140-6736(20)31481-1 32622373 PMC7332261

[53] Tang S Xiang M Cheung T Xiang YT. Mental health and its correlates among children and adolescents during COVID-19 school closure: the importance of parent-child discussion. J Affect Disord. (2021) 279:353–60. 10.1016/j.jad.2020.10.016 33099049 PMC7550131

[54] Wang G Zhang Y Zhao J Zhang J Jiang F. Mitigate the effects of home confinement on children during the COVID-19 outbreak. Lancet. (2020) 395:945–7. 10.1016/S0140-6736(20)30547-X 32145186 PMC7124694

